# The Anti-Inflammatory Effect of Aptamin C on House Dust Mite Extract-Induced Inflammation in Keratinocytes via Regulation of IL-22 and GDNF Production

**DOI:** 10.3390/antiox10060945

**Published:** 2021-06-11

**Authors:** Dahae Lee, Yejin Kim, Hyejung Jo, Cheolhyeon Go, Yoojin Jeong, Yoojin Jang, Dongmin Kang, Kwanjin Park, Yoon-Seong Kim, Jae Seung Kang

**Affiliations:** 1 Laboratory of Vitamin C and Anti-Oxidant Immunology, Department of Anatomy and Cell Biology, Seoul National University College of Medicine, Seoul 03080, Korea; ddhh12345@snu.ac.kr (D.L.); bbambaya921@snu.ac.kr (Y.K.); luv_jo@snu.ac.kr (H.J.); rhcjfgus@snu.ac.kr (C.G.); tlrahrdlf93@snu.ac.kr (Y.J.); pierce52@snu.ac.kr (Y.J.); 2 Medical Research Center, Institute of Allergy and Clinical Immunology, Seoul National University, Seoul 03080, Korea; 3 Department of Psychological and Brain Sciences, College of Arts and Sciences, Boston University, Boston, MA 02215, USA; dong1109@bu.edu; 4 Department of Urology, College of Medicine, Seoul National University, Seoul 16824, Korea; urodori9@snu.ac.kr; 5 Nexmos Co Ltd., U-Tower, 767, Sinsu-ro, Yongin-si 16827, Korea; yk525@rwjms.rutgers.edu

**Keywords:** atopic dermatitis (AD), house dust mites (HDM), vitamin C, Aptamin, Aptamin C, glial cell line-derived neurotrophic factor (GDNF)

## Abstract

Atopic dermatitis (AD), a chronic inflammatory skin disease, is characterized by eczemous lesions on the skin that manifest as severe itching and last a long time. AD is thought to be a response to local allergens, including house dust mites (HDMs). Aptamin C is a modified form of vitamin C comprised of aptamers (DNA fragments) that bind specifically to vitamin C and inhibit its oxidation, thereby increasing its stability and antioxidant effects. It is already known that vitamin C shows an anti-inflammatory effect on skin inflammation. Oxidative stress is one of the major causes of inflammatory diseases, including HDM-induced skin inflammation, suggesting that the antioxidant activity of Aptamin C could regulate inflammatory responses to HDMs in the skin keratinocyte cell line HaCaT and primary skin keratinocytes. Aptamin C not only inhibited HDM-induced proliferation of both type of cells, but suppressed HDM-induced increases in interleukin (IL)-1α and IL-6 production by these cells. In addition, Aptamin C suppressed the production of IL-17 and IL-22 by T cells, which are closely associated with AD pathogenesis, as well as HDM-induced IL-22Rα expression. Aptamin C also reduced the production of thymus and activation-regulated chemokine (TARC) by suppressing the interaction between IL-22 and IL-22Rα, as well as reducing T cell migration. Although HDM treatment markedly increased the expression of glial cell line-derived neurotrophic factor (GDNF), which is associated with itching in AD skin lesions, this increase was reduced by Aptamin C treatment. Taken together, these results suggest that Aptamin C can effectively regulate inflammatory lesions, such as AD, by regulating the production of inflammatory cytokines and GDNF induced by HDM.

## 1. Introduction

Atopic dermatitis (AD) is a chronic inflammatory skin disease associated with itching, dry skin, and eczema. AD is also often accompanied by respiratory diseases, such as allergic rhinitis and asthma. Although the cause of AD has not yet been determined, several factors, including environmental, genetic, and immunological abnormalities, as well as destruction of the skin barrier, are thought to be involved in its pathogenesis. In particular, house dust mite (HDM)-induced keratinocyte dysfunction is associated with skin inflammation in AD; this is because HDM treatment is not only associated with decreases in antimicrobial peptides [1,2,3], but also with increased production of inflammatory chemokines and thymus and activation-regulated chemokine (TARC) [4,5]. 

TARC production is induced by the interaction between interleukin (IL)-22 and its receptor IL-22Rα, followed by migration of T cells into AD skin lesions. IL-22 is a pro-inflammatory cytokine produced mainly by CD4^+^ T cells and natural killer (NK) cells, but it also has anti-inflammatory functions [6,7,8]. IL-22 levels in the skin of AD patients is higher than that in controls, and its production is increased by HDM [9,10,11]. Treatment with IL-22 plus HDM extract induces the production of IL-6, an inflammatory cytokine produced by peripheral blood T cells, in AD patients [4,12,13]. HDM extract also increases the expression of IL-22Rα, a heterodimer consisting of IL-22Rα and IL-10Rβ that is highly expressed in the skin [4]. IL-22Rα plays a pathogenic role in psoriatic skin by inducing differentiation and proliferation of keratinocytes [14,15,16]. Taken together, these findings suggest that IL-22 and its receptor are potential therapeutic targets for regulation of HDM-induced skin inflammation. Thus, new therapeutic agents that regulate the production and expression of IL-22 and its receptor may be very useful for the treatment of HDM-induced skin inflammation in AD.

Nerve growth factor (NGF), released by keratinocytes, is closely related to itching, the most intractable symptom associated with AD skin lesions [17,18]. NGF plays important roles in the survival and differentiation of neurons, as well as in the sensitization of nerve fibers [19,20,21,22,23]. Glial cell line-derived neurotrophic factor (GDNF), a protein produced by glial cells, protects against the degeneration of dopaminergic neurons and increases the number and diameter of nerve fibers with a high affinity for dopamine. Increased production of GDNF in AD skin lesions is a potent cause of itching [24], suggesting that itching in inflammatory AD skin lesions could be regulated by controlling GDNF production. To date, however, no substance that effectively regulates GDNF production has been identified.

Because oxidative stress is a major cause of inflammatory diseases and aging of the skin, antioxidant molecules can effectively protect skin against damage caused by reactive oxygen species (ROS) [25,26,27]. Vitamin C and alloferon exert protective effects against ROS-induced skin inflammation following UVB irradiation [28]. The efficacy of vitamin C may be increased by prolonging its antioxidant activity. Aptamers are single-stranded DNA or RNA oligonucleotides that bind selectively to various molecules. Aptamers that stabilize vitamin C have been identified; indeed, a vitamin C-specific aptamer delays the release of vitamin C, thereby inhibiting its oxidation and prolonging its antioxidant activity [23,29,30]. 

It is already known that vitamin C shows an anti-inflammatory effect on skin inflammation [25,31]. Oxidative stress is one of the major causes of inflammatory diseases [25,27,32], including HDM-induced skin inflammation, suggesting that the antioxidant activity of Aptamin C could regulate inflammatory responses to HDM in the skin keratinocyte cell line HaCaT and primary skin keratinocytes. Therefore, the present study evaluated the effects of Aptamin C on HDM-induced skin inflammation and on changes in inflammation-associated molecules in the HaCaT human keratinocyte cell line, as well as in primary keratinocytes. This study found that Aptamin C downregulates the production of IL-22 and the expression of its receptor by stimulating GDNF production in AD skin lesions, suggesting that Aptamin C, with anti-itching and anti-inflammatory activities, has potential as a therapeutic agent in the treatment of patients with AD.

## 2. Materials and Methods

### 2.1. Cell Culture and Reagent

The HaCaT human keratinocyte cell line, kindly provided by Prof. Kyung Chan Park (Department of Dermatology, Seoul National University College of Medicine), was cultured in RPMI1640 medium (WELGENE, Namcheon-myeon, Korea) supplemented with 10% heat-inactivated fetal bovine serum (FBS; Hyclone, Queensland, Australia) and antibiotics (100 U/mL penicillin and 100 μg/mL streptomycin; WELGENE) at 37 °C in a humidified 5% CO_2_ incubator. 

HDM extract from house dust mites, Dermatophagoides pteronyssinus, was purchased from Cosmo Bio Co. Ltd. (Tokyo, Japan). Mites were homogenized with 10-fold 50 mM phosphate buffer (pH = 7.2), and the homogenate was stirred at 4 °C for 24 h. The centrifugal supernatant was dialyzed four times by PBS (Visking tube, 4 °C).

### 2.2. Isolation and Preparation of Primary Keratinocytes

Foreskin provided by a healthy young male donor was used for the isolation of primary keratinocyte. Skin specimens were incubated overnight at 4 °C in dispase solution (Dispase Ⅱ; Thermo Fisher Scientific, Waltham, MA, USA). The epidermis was separated from the dermis, and epidermal cells were dissociated from the epidermis by treatment with 0.05% trypsin-EDTA (Invitrogen, Carlsbad, CA, USA) for 15 min at 37 °C. After the addition of 10 mg/mL of soybean trypsin inhibitor (Thermo Fisher Scientific), the cells were pelleted, washed, and suspended in keratinocyte serum-free medium (KBM; Thermo Fisher Scientific) containing human keratinocyte growth supplement (Thermo Fisher Scientific) and antibiotics (100 U/mL penicillin and 100 μg/mL streptomycin; WELGENE) at 37 °C in a humidified 5% CO_2_ incubator. Cells used in these experiments were derived from the fourth through the sixth passage of cells grown as a monolayer to subconfluence. Fibroblast contamination of keratinocyte cultures was determined by flow cytometry using a monoclonal anti-human fibroblast antibody (clone ASO_2_). 

All volunteers provided written informed consent. The study protocol was approved by the institutional review board (IRB) of Seoul National University Hospital (IRB No. 2105-095-1219), and all experiments conformed to the principles of the Declaration of Helsinki. 

### 2.3. Reduced Graphene Oxide (rGO)-Based SELEX against Vitamin C

Specific single-stranded DNA (ssDNA) aptamers selectively binding to vitamin C were produced using the systematic evolution of ligands by the exponential enrichment (SELEX) method. An ssDNA aptamer library consisting of 30 randomly-generated nucleotide sequences of 60 nucleotides each, flanked by primer sites for amplification (5′-ATGCGGATCCCGCGC-(N)30-GCGCGAAGCTTGCGC-3′), was generated [33]. The ssDNA aptamer library was subjected to five rounds of enrichment, and the fourth round included a counter selection process in an attempt to obtain more specified sequences. 

The ssDNA library-bound rGO was prepared in 1× binding reaction buffer (135 mM of NaCl, 2.7 mM of KCl, 4.3 mM of Na_2_HPO_4_, 1.4 mM of KH_2_PO_4_, 1 mM of MgCl_2_), followed by incubatation for 30 min in order to immobilize the ssDNA library onto the rGO. The mixture was centrifuged at 13,000 rpm for 15 min, and the remaining unbounded ssDNA was removed in response to the elimination of the supernatant. The ssDNA library-bound rGO pellet was first rinsed with 200 µL of 1× binding buffer, then re-cetrifuged under the same environment in order to further eradicate any remaining unbounded ssDNA. Then, 40 nanomoles of vitamin C, adulterated in 200 µL of 1× binding buffer, were added to the pellet. To elute the target binding aptamer from the rGO, vitamin C and the rGO mixture were incubated for 1 h. The eluted candidates were duplicated via the same process incorporated in the construction of the ssDNA library. A course of this series was regarded as a single round. In order to select the adequeate sequence for the target, the binding time and buffer concentration were regulated in each round.

### 2.4. NXP031 Preparation

DNA aptamers were purchased from Integrated DNA Technologies. They were dissolved in PBS that contained 1 mM of MgCl_2_ and heated for 5 min at 95 °C. They were then allowed to cool to room temperature to enable the aptamers to fold into their tertiary structures. L-ascorbic acid (Sigma Aldrich, St. Louis, MO, USA) was added to the DNA aptamer at a ratio of 1:50 (*w*/*w*).

### 2.5. Confocal Microscopy

HaCaT and primary keratinocyte cells were grown on 1 cm^2^ cover slips at 37 °C under a 5% CO_2_ atmosphere for 24 h. After washing with PBS, HaCaT was treated with HDM extract (25 μg/mL), TNF-α, and IFN-γ (10 ng/mL), and cultured for 24 h. The HaCaT grown on the cover slips was fixed in 4% paraformaldehyde (PFA) and pre-incubated overnight with 5% goat serum in PBS-T (0.3% Triton X-100 in PBS). Cells were incubatd with rabbit anti-human IL-22Rα antibody (Abcam, Cambridge, UK) and an Alexa Fluor 633-conjugated anti-rabbit antibody (Invitrogen). The fluorescence images were captured under an inverted confocal microscope (FV3000; Olympus, Tokyo, Japan) after mounting with a DAPI-containing mounting solution (Immunobioscience, Mukilteo, WA, USA).

### 2.6. Isolation of T Cells

Under the protocol approved by the Seoul National University Hospital IRB (#1507-117-690), the peripheral blood mononuclear cells (PBMCs) were obtained from healthy adult donors. T cells were purified from PBMCs using a MACS negative selection system and a Pan T cell isolation kit (Miltenyi Biotec, Bergisch Gladbach, Germany). The purity of the T cell was determined by staining the cell with an APC-conjugated anti-CD3 antibody (e-Bioscience, San Diego, CA, USA), followed by flow cytometry and data analysis using FlowJo software (Tree Star, Ashland, OR, USA).

### 2.7. Enzyme-Linked Immunosorbent Assay (ELISA) 

Human T cells, HaCaT, and the primary keratinocyte cells were treated with HDM extract (25 μg/mL) for 24, 48, or 72 h, accordingly, and the concentrations of TNF-α, IFN-γ, IL-22, and GDNF in the cell supernatants were evaluated via ELISA by referring to the manufacturer’s instructions (R&D Systems). The optical density of each well was determined by using SoftmaxPro software (Molecular Devices). HaCaT was seeded either with or without HDM extract (25 μg/mL), followed by treatment with culture supernatants from T cells, human IL-22 antibody (R&D)-treated T cells, or HDM extract-treated T cells. The supernatants were harvested after 24, 48, or 72 h, accordingly, and the concentrations of TARC, IL-1α, and IL-6 in the culture supernatants were evaluated via ELISA by referring to the manufacturer’s instructions (Biolegend, San Diego, CA, USA). Relative absorbance was measured at 450 nm, and concentrations were calculated using the SpectraMac iD3 (Molecular Devices).

### 2.8. Transwell Migration Assay 

Transwell migration assays were performed by using a microchamber Transwell system with 5 μm pores (Corning Costar, Tewksbury, MA, USA). Supplemented with 1% heat-inactivated FBS, human T cells in RPMI1640 medium were added to each upper compartment of the insert, and the HDM extract (25 μg/mL) or culture supernatants from HDM extract (25 μg/mL)-treated HaCaT (48 and 72 h) were added to each lower compartment. T cells were allowed to migrate to the lower compartment for 3 h. The upper compartment was removed, and the T cells in the lower compartment were evaluated and expressed as a percentage of the total number of T cells added to the upper well.

### 2.9. Gene Expression Profiling

Gene expression by HaCaT treated with HDM, vitamin C, aptamin, or Aptamin C was analyzed using Affymetrix GeneChip^®^ Human Gene 2.0 ST arrays. Total cellular RNA was extracted from 1 × 10^6^ cells using TRIzol (Invitrogen). RNA quality was assessed by an Agilent 2100 Bioanalyzer using the RNA 6000 Nano Chip (Agilent Technologies, Santa Clara, CA, USA), and quantity was determined using a Nanodrop-1000 Sepctrophotometer (Thermo Scientific). A 300 ng aliquot of each RNA sample was subjected to the Affymetrix procedure, as recommended by the manufacturer. The DEGs were functionally annotated by using the web-based tool Database for Annotation, Visualization, and Integrated Discovery (DAVID), and were classidied based on the information regarding the gene function, which included basic statistics, hierarchical clustering, K means, *t*-tests, and analysis of variance (ANOVA). To identify the regulatory networks, the integrated analysis (mRNA/miRNA) databases were analyzed.

### 2.10. Statistical Analysis

Data were expressed as the mean ± SD and compared between two groups using unpaired two-tailed *t*-tests. Comparisons between three or more groups were made using one-way ANOVA, followed by the Newman–Keuls multiple comparisons test; *p*-values < 0.05 were considered statistically significant. All statistical tests were performed using GraphPad InStat (GraphPad Software, San Diego, CA, USA).

## 3. Results

### 3.1. Aptamin C Suppresses HDM-Induced Proliferation of HaCaT and Primary Human Keratinocytes

The proliferation of keratinocytes is closely associated with inflammatory responses in skin diseases, including psoriasis and AD [16,34]. In addition, HDM extract induces skin inflammation and proliferation of the HaCaT skin keratinocyte cell line. Therefore, we assessed whether Aptamin C suppresses HDM-induced proliferation of HaCaT and primary human keratinocytes. We found that both vitamin C and Aptamin C effectively suppressed proliferation of HaCaT and human primary keratinocytes induced by HDM extract (Figure S1), with Aptamin C having a greater effect than vitamin C. 

### 3.2. Aptamin C Suppresses HDM-Induced Production of IL-1α and IL-6 by HaCaT and Primary Keratinocytes

**Figure 1:**
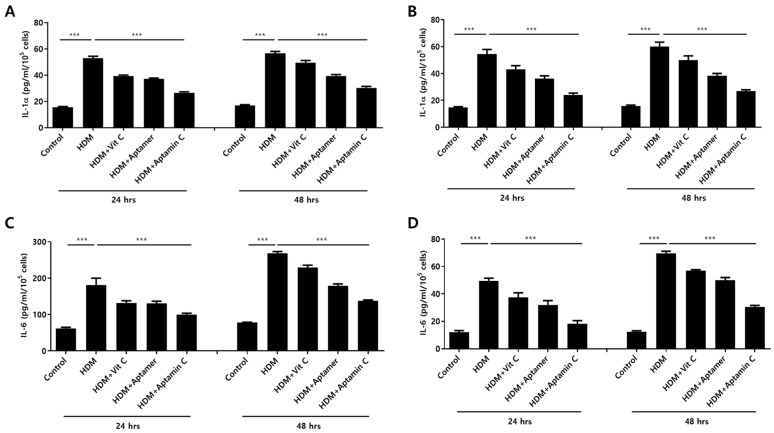
Effects of HDM extract on IL-1α and IL-6 production by HaCaT and primary keratinocytes, and suppression by Aptamin C. HaCaT (**A**,**C**) and primary human keratinocytes (**B**,**D**) were treated with 25 μg/mL of HDM extract, with or without vitamin C (40 μM), Aptamin (0.09 μg/mL), or Aptamin C (vitamin C 40 μM/Aptamin 0.09 μg/mL) for 24 or 48 h. The culture supernatants were collected, and the concentrations of IL-1α (**A**,**B**) and IL-6 (**C**,**D**) were evaluated by ELISA as described in Materials and Methods. Data are presented as the mean ± SD. *** *p* < 0.001.

HDM extract also increases IL-1α and IL-6 production by HaCaT. Therefore, we assessed whether Aptamin C could suppress HDM-induced IL-1α and IL-6 production by HaCaT and primary keratinocytes. The production of IL-1α and IL-6 by HaCaT and primary keratinocytes was markedly enhanced by treatment with HDM extract, but this production was effectively suppressed by treatment with vitamin C, Aptamin, and Aptamin C (Figure 1). 

### 3.3. Aptamin C Suppresses HDM-Induced IL-17 and IL-22 Production by T Cells

**Figure 2:**
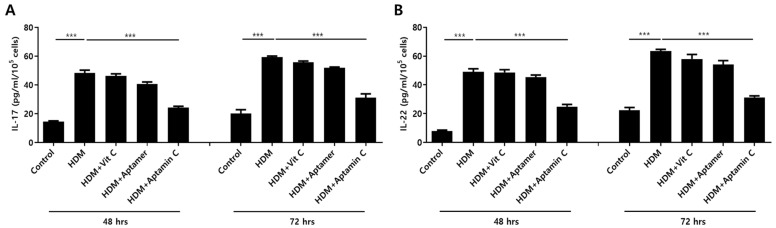
Effects of HDM extract on T cell production of IL-17 and IL-22, and suppression by Aptamin C. T cells isolated from peripheral blood mononuclear cells (PBMCs) and purified with a MACS negative selection system, as described in Materials and Methods, and then treated with 25 μg/mL of HDM extract, with or without vitamin C (40 μM), Aptamin (0.09 μg/mL), or Aptamin C (Vitamin C 40 μM/Aptamin 0.09 μg/mL) for 48 or 72 h. The culture supernatants were collected, and the concentrations of IL-17 (**A**) and IL-22 (**B**) were measured by ELISA. Data are presented as the mean ± SD. *** *p* < 0.001.

IL-17 and IL-22 are closely associated with the pathogenesis of skin inflammation, especially AD [7,35,36]. Therefore, we examined the ability of Aptamin C to regulate the expression of HDM-induced IL-17 and IL-22 production by human peripheral T cells. Exposure of T cells purified from human PBMCs to HDM extract in the presence or absence of vitamin C, Aptamin, or Aptamin C showed that HDM extract increased IL-17 and IL-22 production, and that these increases were effectively suppressed by Aptamin C (Figure 2). 

### 3.4. Aptamin C Suppresses HDM-Induced IL-22Rα Expression by HaCaT and Primary Keratinocytes

**Figure 3:**
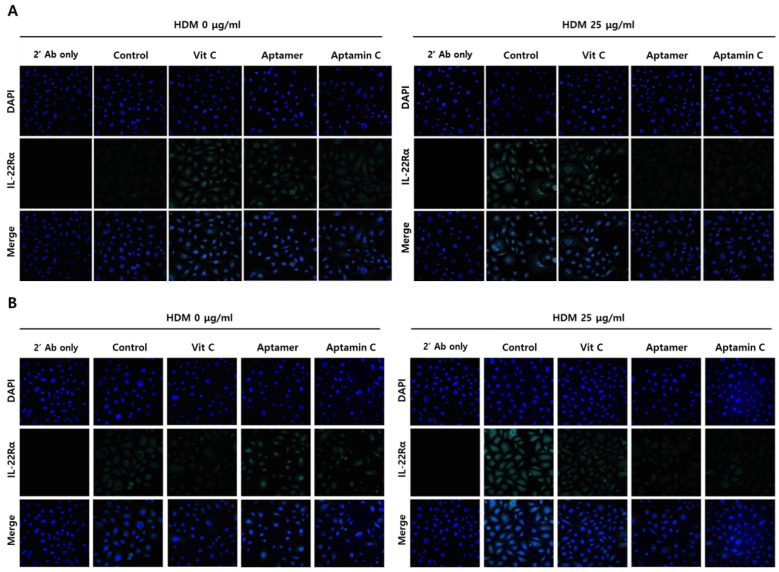
Effects of HDM extract on IL-22Rα expression by HaCaT and primary keratinocytes, and suppression by Aptamin C. (**A**) HaCaT and (**B**) primary keratinocytes were pre-treated with HDM (25 μg/mL) for 24 h, fixed with 4% paraformaldehyde (PFA), and pre-incubated with 5% goat serum in PBS-T. The cells were incubated with anti-human IL-22Rα antibody (1:150) or vehicle and with Alexa Fluor 633-conjugated anti-rabbit secondary antibody (1:1500), followed by nuclear staining with 4’,6-diamidino-2-phenylindole (DAPI; blue). Fluorescence microscopic images were captured and analyzed by confocal microscopy. Results are the representative of three independent experiments.

Because IL-22Rα plays a role in HDM-induced skin inflammation, we assessed whether Aptamin C could regulate HDM-induced IL-22Rα expression by HaCaT and primary keratinocytes. Immunofluorescence staining showed that HDM extract markedly enhanced the expression of IL-22Rα on HaCaT and primary keratinocytes, but this enhancement was suppressed by treatment with Aptamin C (Figure 3). Interestingly, vitamin C could not regulate IL-22Rα expression, whereas Aptamin could.

### 3.5. Aptamin C Suppresses TARC Production by HaCaT and Primary Keratinocytes, and Suppresses T Cell Migration

**Figure 4:**
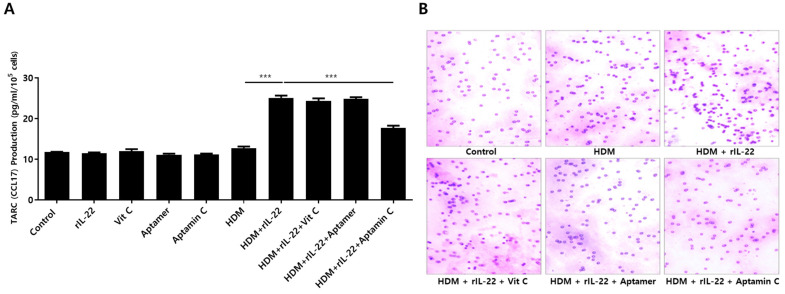
Effects of HDM extract on thymus and activation-regulated chemokine (TARC) production by HaCaT and on T cell migration, and suppression of these effects by Aptamin C. (**A**) Aptamin C downregulates the HDM extract-associated increase in production of TARC by HaCaT. Data are presented as the mean ± SD. *** *p* < 0.001. (**B**) Transwell migration assays showing Aptamin C-associated suppression of HDM extract-enhanced T cell migration over 48 or 72 h. Results are the representative of three independent experiments.

TARC is associated with chemotaxis of T cells, particularly Th2 cells, and is involved in the pathogenesis of skin inflammation in AD by enhancing migration of T cells into skin lesions [4,37]. Additionally, TARC production results from the interaction between IL-22 and its receptor on skin keratinocytes [6,12]. Because Aptamin C downregulates HDM-induced IL-22 production by T cells, as well as IL-22Rα expression by HaCaT, we assessed the effects of Aptamin C on TARC production. As expected, HDM extract increased TARC production by HaCaT, an increase effectively downregulated by treatment with Aptamin C (Figure 4A). This decrease in TARC production was accompanied by a decrease in T cell migration (Figure 4B). 

### 3.6. Aptamin C Suppresses HDM-Induced GDNF Production by HaCaT and Primary Keratinocytes

**Figure 5:**
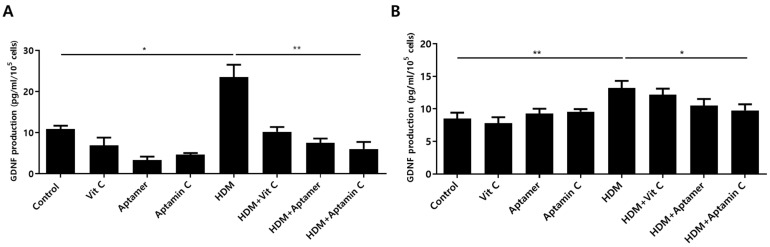
Effects of HDM extract on GDNF production by HaCaT and primary keratinocytes, and suppression by Aptamin C. HaCaT (**A**) and primary human keratinocytes (**B**) were treated with 25 μg/mL of HDM extract, with or without vitamin C, Aptamin, or Aptamin C, for 48 h. Culture supernatants were collected, and the concentrations of GDNF were measured by ELISA. Data are presented as the mean ± SD. * *p* < 0.05, ** *p* < 0.01.

Factors altered in HaCaT and primary keratinocytes by treatment with HDM extract and Aptamin C were analyzed using the Human Gene 2.0 ST array. The expression of 31 genes was increased by treatment with HDM extract, but decreased by treatment with Aptamin C (Supplementary Table S1). Among these changes were alterations in the expression of mRNA-encoding glial cell line-derived neurotrophic factor (GDNF), the major causative factor for itching during skin inflammation in AD [24,38]. To assess whether HDM extract and Aptamin C induced similar changes in GDNF protein expression, the concentrations of GDNF protein in cell supernatants were assayed by ELISA. Treatment of HaCaT and primary keratinocytes with HDM extract markedly increased GDNF production, an increase effectively suppressed by treatment with Aptamin C (Figure 5).

## 4. Discussion

AD is a common chronic inflammatory skin disease characterized by pruritis, redness, and eczema. The causes of AD vary widely, and can include both genetic and environmental factors [39,40]. HDM is an environmental factor frequently associated with the development of AD, but few studies to date have analyzed substances that regulate the development of HDM-associated AD [4,11,41]. Our previous study showed that treatment with HDM extract increases IL-22Rα expression and TARC production by T cells, as well as enhances IL-22 production and migration by T cells [4,42,43]. The present study expands these findings by analyzing substances that may regulate HDM-induced inflammatory responses in skin keratinocytes and T cells.

Aptamin C is a substance that combines vitamin C with an aptamer, a DNA fragment that binds specifically to vitamin C. Vitamin C-specific aptamers enhance the stability of vitamin C by preventing its rapid oxidation through interactions with oxygen or aqueous solutions [44]. Moreover, Aptamin C increases skin moisturization and improves pruritus [30]; it also showed a neuroprotective effect in an animal model of Parkinson’s disease induced by the neurotoxin 1-methyl-4-phenyl-1,2,3,6-tetrahydropyridine (MPTP) [23]. The development and progression of skin diseases, including AD and psoriasis, are closely associated with ROS-mediated immune responses, and the antioxidant vitamin C is an effective anti-inflammatory molecule, because it acts as an ROS scavenger [45]. Thus, Aptamin C may be a promising candidate for regulating HDM-induced skin inflammation and for the treatment of AD.

Inflammatory responses in the skin are associated with the proliferation of skin keratinocytes; as such, they are induced by HDM extract [4,46,47,48]. Thus, substances that regulate HDM extract-induced proliferation of keratinocytes may also be useful for regulating inflammation in skin diseases, such as AD. As expected, Aptamin C effectively regulates HDM extract-induced proliferation of the HaCaT skin keratinocyte cell line, as well as primary skin keratinocytes. Because psoriasis is caused by the proliferation of keratinocyte, Aptamin C may be an effective treatment. To confirm this, research is underway to examine its effects in animal models of psoriasis, including imiquimod-induced psoriasis, and in 3D skin models based on primary human keratinocytes.

Keratinocytes are involved in birth immunity, as they form the skin barrier, while releasing a variety of cytokines, such as IL-1α and IL-6, which play important roles in inducing immune responses in the skin [28,49]. HDM extract-mediated induction of excess inflammatory cytokines, such as IL-1α and IL-6, by keratinocytes can lead to serious inflammatory responses in the skin [4,11,28,49]. Therefore, Aptamin C-mediated inhibition of keratinocyte proliferation not only regulates inflammatory responses in the skin through the suppression of increased production of IL-1α and IL-6, but also through the direct suppression of the production of these cytokines by keratinocytes.

IL-22 is involved in the induction of keratinocyte migration and pro-inflammatory gene expression, as well as in the induction of proliferation of normal human epidermal keratinocytes [28,50,51]. IL-22 production in skin lesions in animal models of AD and in AD patients is higher than in respectively normal controls [41,49,52,53]. Moreover, IL-6 production by peripheral blood T cells is higher in AD patients than in control subjects [54]. Co-treatment of keratinocytes with HDM extract and rIL-22 increases IL-6 production. IL-22 is produced by CD3-c-Kit+ cells in skin lesions of patients with psoriasis and AD, and c-Kit + FcεRI+ mast cells are the major cellular source of IL-22 in AD patients [53]. Downregulation of IL-22Rα expression may control inflammatory processes in skin lesions of patients with psoriasis and AD. IL-22R consists of IL-22Rα and IL-10Rβ, with an increase in IL-22Rα expression, which is regarded as a hallmark of inflammation, especially in the skin [26,51]. HDM extract increases IL-22Rα expression on keratinocytes [4,11,46]. Moreover, IL-22Rα is closely involved in inflammatory processes in the skin through interaction with IL-22, the expression of which by T cells is enhanced by HDM extract (4). Increased IL-22Rα expression on both HaCaT and primary keratinocytes was suppressed by treatment with Aptamin C, strongly suggesting that Aptamin C effectively regulates IL-22-mediated skin inflammation not only by suppressing T cell production of IL-22, but also by suppressing keratinocyte expression of IL-22Rα.

In addition to being involved in the production of pro-inflammatory cytokines, the interactions between IL-22 and IL-22Rα play an important role in T cell migration by increasing the production of TARC, a chemokine that plays a pivotal role in AD pathogenesis. TARC production is higher in AD patients and AD animal model NC/NgA mice than in the corresponding normal controls. Keratinocytes are the major cellular source of TARC, and TARC production by these cells is increased by HDM extract [4,5,42,55]. TARC promotes T cell migration [43,56,57]. The present study found that Aptamin C suppresses the increase in TARC expression induced by treatment with HDM extract and rIL-22. Aptamin C also effectively suppressed T cell migration that was increased by the culture supernatant of HDM extract-treated HaCaT in the presence of rIL-22. Therefore, Aptamin C inhibits HDM-induced IL-22 production by T cells while inhibiting the migration of IL-22-producing T cells into skin lesions.

Molecular analysis of the effects of HDM, with or without Aptamin C, on gene expression by HaCaT and primary keratinocytes identified 31 genes, the expression of which was increased significantly by treatment with HDM extract, but decreased significantly by treatment with Aptamin C. One of these genes encodes GDNF, originally identified as enhancing the survival of neurons through increased dopamine uptake [58,59], but was recently reported to be closely associated with inflammation, especially with severe itching in AD [38,58]. In addition, artemin, a member of the GDNF family, is involved in hypersensitivity to warm sensations, mimicking warmth-provoked pruritus in AD [60]. Therefore, we assessed whether HDM extract could increase GDNF production by HaCaT and primary keratinocytes, and whether these increases could be suppressed by Aptamin C. As expected, HDM extract markedly increased the production of GDNF protein, an increase suppressed by treatment with Aptamin C. A cream containing Aptamin C has skin moisturizing effects and reduces itching in ordinary people with dry skin. Ongoing clinical studies suggest that an ointment containing Aptamin C effectively regulates pruritus in patients with AD.

The present study reports the anti-inflammatory effects of Aptamin C on skin inflammation and its related mechanisms. Taken together, the findings indicate that Aptamin C may be a candidate for the treatment of skin inflammation, especially itching. Because Aptamin C increases the stability of vitamin C, Aptamin C may facilitate greater improvements in diseases treated effectively with vitamin C.

## 5. Conclusions

**Figure 6:**
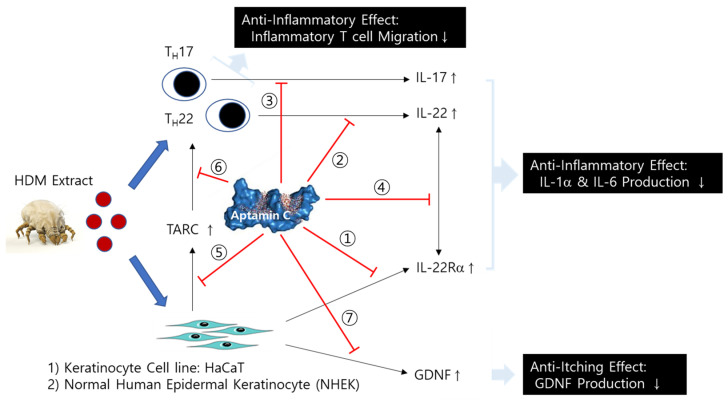
Mechanism of the anti-inflammatory and anti-itching effect of Aptamin C on skin inflammation by HDM. Aptamin C shows an anti-inflammatory effect by the suppression of ① IL-22Rα expression on HaCaT, ② IL-22 production from T cells, ③ IL-17 production from T cells, ④ the interaction between IL-22 and IL-22Rα, ⑤ TARC production via the interaction between IL-22 and IL-22Rα, and ⑥ TARC-induced T cell migration, as well as an anti-itching effect by the suppression of ⑦ GDNF production from HaCaT and primary keratinocytes.

Aptamin C more effectively regulates HDM-induced inflammatory responses than vitamin C in the skin keratinocyte cell line, HaCaT, and primary keratinocytes via the suppression of (1) IL-22Rα expression on HaCaT, (2) IL-22 production from T cells, (3) IL-17 production from T cells, (4) the interaction between IL-22 and IL-22Rα, (5) TARC production via the interaction between IL-22 and IL-22Rα, and (6) TARC-induced T cell migration (Figure 6). It also shows anti-itching effects by the suppression of GDNF production from HaCaT and primary keratinocytes (Figure 6). Therefore, Aptamin C is a useful substance for skin inflammatory diseases, such as atopy dermatitis (AD) and psoriasis.

## Data Availability

The data generated during this study are included in this article and are available on request from the corresponding author.

## Supplementary Materials

The following are available online at https://www.mdpi.com/article/10.3390/antiox10060945/s1, Supplementary Figure S1. Effects of HDM extract on proliferation of HaCaT. Supplementary Table S1. Gene expression profiling. The table and heatmap display individual differentially expressed genes, and the table depicts a subset of the genes with the largest mean Log_2_ fold-changes when comparing HDM-treated and control samples.
