# Supplementary material for: The Anti-Inflammatory Effect of Aptamin C on House Dust Mite Extract-Induced Inflammation in Keratinocytes via Regulation of IL-22 and GDNF Production
Source: Antioxidants (Basel). 2021 Jun 11;10(6):945. doi: 10.3390/antiox10060945 (PMC8230602; doi:10.3390/antiox10060945)
Supplement: Supplementary file 1 [file antioxidants-10-00945-s001.zip › antioxidants-1240090-supplementary.pdf]

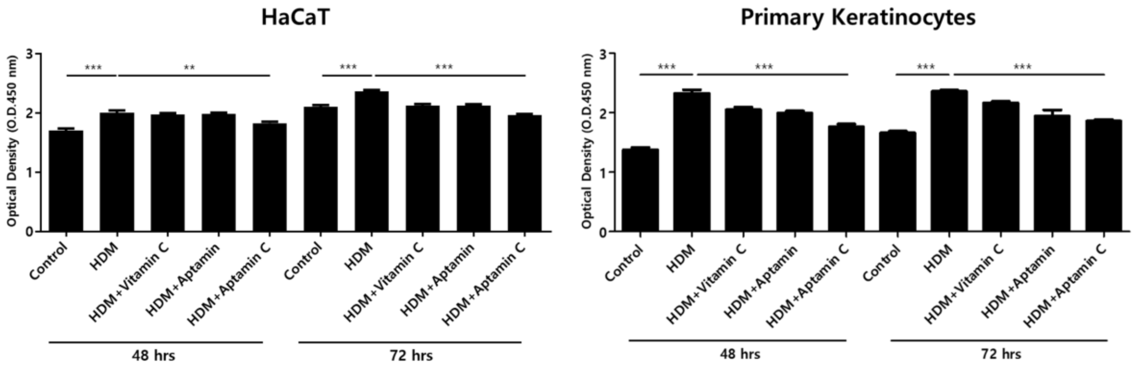

Figure 1. Proliferation of HaCaT and human keratinocytes induced by HDM extract was effectively suppressed by the treatment of Aptamin C.

	Sample ID	Sample Group	Cel Files
1	CTRL	CTRL	210106_13_CTRL_(HuGene-2_0-st).CEL
2	VC	VC	210106_14_VitaminC_(HuGene-2_0-st).CEL
3	AP	AP	210106_15_Aptamin_(HuGene-2_0-st).CEL
4	VC_AP	VC_AP	210106_16_VitaminC_Aptamin_(HuGene-2_0-st).CEL
5	HDM	HDM	210106_17_HDM_(HuGene-2_0-st).CEL
6	HDM_VC	HDM_VC	210106_18_HDM_VitaminC_(HuGene-2_0-st).CEL
7	HDM_AP	HDM_AP	210106_19_HDM_Aptamin_(HuGene-2_0-st).CEL
8	HDM_VC_AP	HDM_VC_AP	210106_20_HDM_VitaminC_Aptamin_(HuGene-2_0-st).CEL

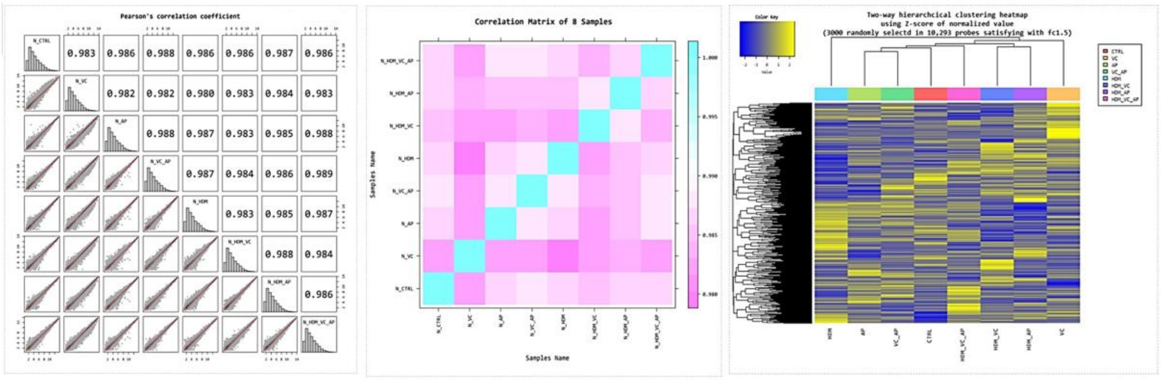

Figure 2. Gene expression profiling. Table and heatmap displays individual differentially expressed genes and the table depicts a subset of the largest Log_2 fold-change within HDM-induced sample verses control sample Log_2 fold-change mean values.
